# Social disparities in children’s exposure to second hand smoke at home: a repeated cross-sectional survey

**DOI:** 10.1186/1476-069X-11-65

**Published:** 2012-09-17

**Authors:** Charlotta Pisinger, Lene Hammer-Helmich, Anne Helms Andreasen, Torben Jørgensen, Charlotte Glümer

**Affiliations:** 1 Research Centre for Prevention and Health, The Capital Region of Denmark, Glostrup, Denmark; 2 Faculty of Health Science, University of Copenhagen, Copenhagen, Denmark; 3 Faculty of Medicine, University of Aalborg, Aalborg, Denmark

**Keywords:** Second hand smoke, Environmental tobacco smoke pollution, Involuntary smoking, Passive smoking, Child, Child welfare, Social classes, Socioeconomic status

## Abstract

**Background:**

Exposure to second hand smoke (SHS) is an important preventable cause of morbidity and mortality in children. We hypothesised that there has been a growth in social inequality in children’s exposure to SHS at home over time. The purpose of this study was to investigate temporal change in smoking in homes including children, focusing on the socioeconomic differences.

**Methods:**

Data is from a repeated cross-sectional survey, ‘Health Profiles of the Capital Region of Denmark’ conducted in 2007 and 2010, in 29 municipalities. The response rate was 52.3%, in both surveys. Our study included persons aged 25 to 64, living with children ≤15 years of age; N=9,289 in 2007 and 12,696 in 2010. Analyses were weighted for size of municipality and for non-response, which was higher among men and among persons who were younger, had a lower income, were living alone, were unemployed, and/or were of an ethnicity other than Danish. Regression analyses were used to investigate smoking in homes including children across parent/adult education levels, focusing on temporal changes.

**Results:**

There were significant large socioeconomic differences in both 2007 and 2010. In 2010 it was more than 11 times more likely for a child to be exposed to SHS at home if the parent had very low education than if they were highly educated (p<0.001). Smoking in a home with children decreased from 16.2% in 2007 to 10.9% in 2010. The odds of a temporal decrease in domestic smoking did not differ significantly across parent education levels (p=0.40).

**Conclusions:**

Marked social inequalities in children’s exposure to SHS and a significant temporal decrease in exposure, independent of the education level of the parent/adult, were found in repeated large cross-sectional population-based studies. Social disparities have persisted over time, but not increased. Both clinical and population policy interventions will be needed in order to control child SHS exposure. We call for legislative protection of children from tobacco smoke inside their home as a supplement to parental education and support for smoking cessation.

## Background

Exposure to second hand smoke (SHS) at home is an important preventable cause of morbidity and mortality in children. The evidence for a causal relationship between SHS and asthma induction and exacerbation, respiratory-and middle ear infections, reduced lung function and sudden infant death syndrome is quite strong [1-4] The impact of postnatal SHS exposure on childhood cancer, deficits in intellectual ability, and behavioural problems remains less clear, but is a matter of concern [5-8]. Exposure to tobacco smoke in children is also associated with attenuated endothelial function and reduced flow-mediated dilatation, early precursors to heart disease in later life, suggesting irreversible impairment of endothelium-dependent vasodilatation [9,10]. Children’s exposure to SHS is also associated with many other adverse health effects, such as being overweight, insulin resistance, a higher prevalence of caries and hearing loss [11-14].

Not only does smoking at home have a dramatic influence on the exposed child, it also places a heavy burden on society. It has been estimated that the annual cost of SHS that results in childhood illness and death is many billions of dollars [15,16].

Exposure to passive smoking among children has been significantly reduced in western countries over recent decades, which can partly be explained by a fall in the percentage of mothers and fathers who smoke [17-19]. However, there has also been a secular trend towards smoke-free homes, even when parents themselves are smokers [20,21].

Children’s exposure to SHS is known to vary according to their parents’ socioeconomic status (SES) [22,23]. A lower socioeconomic status is strongly associated with higher smoking rates, and the steep social class gradient in smoking has worsened over the past decades [24].

One could hypothesise that there has been a growth in social inequality over time, as persons with higher education implement smoke-free homes more frequently than those with low education. Two recent studies from Wales and Australia, have found an increasing gap in social inequality in children’s exposure to SHS [25,26].

In the Eurobarometer survey 2010 Denmark was one of the most permissive European countries when it comes to SHS exposure. Only one in two adults in the survey stated that “Smoking is not allowed at all inside the house”, compared with nine out of ten in neighbouring Finland and Sweden [27]. Little is known about children’s exposure to SHS in Denmark, and no previous peer-reviewed study has investigated social inequality in children’s exposure to SHS over time.

The aim of this study was to investigate temporal changes in smoking in homes with children in the Capital Region of Denmark, through large population-based surveys, focusing on socioeconomic differences.

## Methods

The Capital Region, consisting of 29 municipalities, is the largest region of Denmark, with approximately 1.2 million inhabitants aged 25 years or older.

The data used was taken from an independent cross-sectional survey, conducted in 2007 and 2010, the ‘Health Profiles of the Capital Region’. Random samples of all citizens were drawn from the Civil Registration System using computer generated random numbers. Due to population size, the City of Copenhagen municipality was divided into ten units and each unit was treated as one municipality for the sampling process.

The first survey was conducted in September 2007 (except in five municipalities where the survey was performed in 2006). In each of the 29 municipalities a random sample of 1,600 to 1,800 adult persons received a mailed questionnaire, “How are you?” containing questions regarding their lifestyle/health-related behaviour, general health and chronic diseases.

The second survey was conducted in February 2010. In each municipality a new random sample of at least 2,450 adult persons received a mailed questionnaire of the same size and with almost the same content. The response rate was 52.3%, in both surveys; 36,472 questionnaires were returned in 2007 and 49,806 in 2010.

In Denmark every person is given a personal and permanent 10-digit identification number at birth or on immigration, which is used to register all information about mortality, disease diagnosis, socio economic status etc. in a central register. The registers have a high validity. Information on age, sex and education was obtained from the National Central Registers.

The research project was approved by the Danish Data Protection Agency according to the Danish Act on Processing of Personal Data. Approval from the Danish Health Research Ethics Committee System was not required according to Danish law, as the research project was purely based on data from questionnaires and registers. Written informed consent for publication of data was given from the participants when returning the questionnaires.

### Persons included in this paper

**Figure 1:**
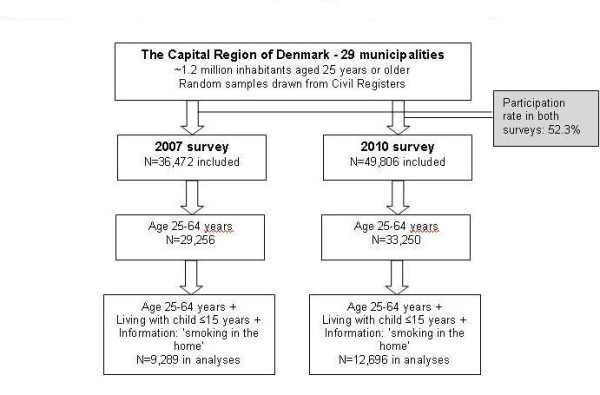
Number of persons included inhe two surveys and in the analyses.

This paper includes participants of the survey, smokers and non-smokers, who completed the question “Does smoking take place indoors in your home?”. Smoking in the home was dichotomised to two categories: 1) no (never or almost never/less than weekly) and 2) yes (weekly/daily). Additionally, responders should be between 25 and 64 years old and living with child/children aged 15 years or less. This age limit was chosen because it was the lowest age in the 2007 survey; the highest age limit was chosen because very few participants over the age of 64 have children aged 15 years or less living at home. A total of 9,289 participants were included in 2007 and 12,696 in 2010 (Figure 1).

### Definition of ‘parent’

As information about cohabiting with a child/children comes from the question “How many children aged 15 or below live in your household?” we do not know whether the responding adult is the child’s parent, step-parent, older sibling, guardian or grandparent. However, almost all Danish children move away from home after finishing basic education, there is no tradition for cohabiting with grandparents or other relatives and few children have guardians. It is therefore assumed that, in almost all cases, the adult is a parent or step-parent, and for the sake of convenience we call the adult responders cohabiting with children ‘parents’ in the manuscript.

### Socioeconomic status

Information about the highest level of education completed by participants in the survey (not the person with the highest education in the family) was obtained from the Danish Population’s Education Register (PER). The resulting variable was categorized as either very low education (i.e. primary school only, e.g. unskilled worker), low education (i.e. up to two years of vocational training, e.g. carpenter, hair-dresser), medium education (i.e. more than two years and less than four years of education e.g. teacher, policeman) and high education (i.e. four years or more of academic education, e.g. doctor, psychologist).

### Statistics

All analyses were weighted due to the complex sampling design of the survey. They were weighted for size of municipality, as citizens living in a large municipality were less likely to be selected, and for non-response which was higher among men and among persons who were younger, had a lower income, were living alone, were unemployed, or/and were of an ethnicity other than Danish. The results are therefore not only representative for the whole random sample but also for the population in every municipality, in spite of differences in participation across municipalities and population groups. The weights were computed by Statistics Denmark based on registry information about sex, age, municipality of residence, highest completed education level, income, civil status and hospitalization for all individuals invited to the survey.

Logistic regression analyses were used to investigate whether there were differences from 2007 to 2010 in smoking in homes with children, adjusting for the parent’s sex, age and education. Using the same regression analyses we investigated differences in smoking at home across parent education level and whether temporal changes differed across education level. Models were adjusted for sex and age, and tested for interaction between time and sex, time and age, and time and education. Finally, the analyses were repeated, controlling for parent’s smoking status and testing for interaction between time and smoking status. However, as we believe that a person’s smoking status is a mediator, and not a confounder, for exposure to SHS, we do not show these results as final results, but offer them for discussion only.

Statistical analyses were performed using SAS statistical software (version 9.3, SAS Institute Inc., Cary, NC, USA).

## Results

Self-reported smoking prevalence in the selected age-groups 25 to 64 in the Capital of Region of Denmark decreased from 22% in 2007 to 19% in 2010, and the proportion of smokers with high daily tobacco consumption decreased at the same time (Table 1).

**Table 1:** Baseline characteristics of the study population in 2007 and 2012

	**2007**		**2010**	
	**N**	**%**	**N**	**%**
Sex=male	29,256	44.02	33,250	43.59
Age	29,256		33,250	
25-34 years		19.74		18.57
35-44 years		26.57		25.95
45-54 years		25.26		27.11
55-64 years		28.42		28.37
Daily smoker=yes	29,041	22.07	32,610	19.16
Tobacco consumption, daily smokers	6,379		6,224	
Light smoker (<15 cigarettes daily)		38.14		43.88
Heavy smoker (≥15 cigarettes daily)		61.86		56.12
Education	28,584		32,387	
Very low		15.19		14.47
Low		47.60		46.81
Medium		22.71		22.85
High		14.50		15.86

In the same time period smoking in homes with a child/children decreased from 16.2% to 10.9%. In regression analyses, adjusted for sex, age and education of the parent the odds ratio for smoking in a home with children in 2010 was 0.63 (95%CI: 0.58-0.70) compared with 2007. Domestic smoking and exposure of children to SHS was significantly less frequent in homes where the parent was a non-smoker; odds ratio: 0.065 (95%CI: 0.06-0.07).

**Figure 2:**
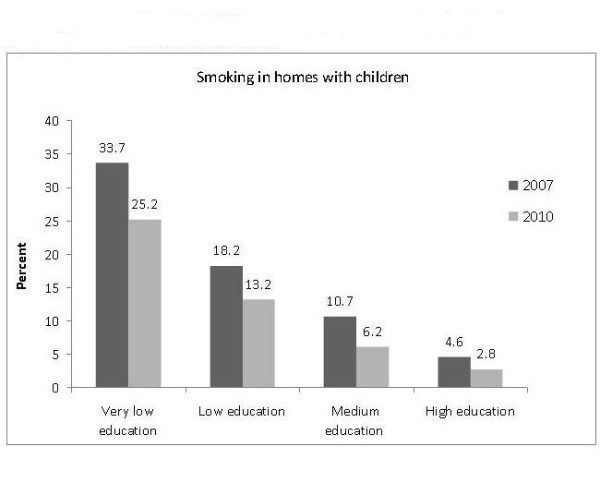
**Smoking inside homes with children.** Differences across parents’ education level. Capital Region of Denmark in 2007 and 2010.

**Figure 3:**
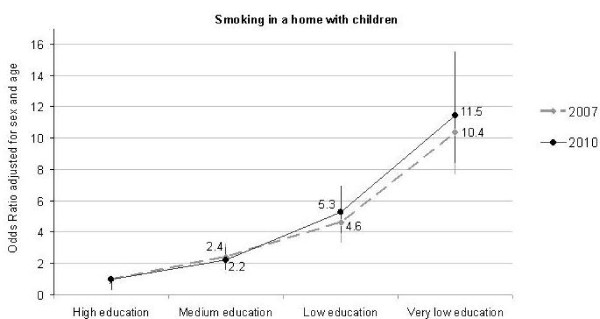
**Smoking inside homes with children by parents level of education in 2007 and 2010.** Analyses adjusted for age and sex. Capital Region of Denmark.

There were large socioeconomic differences in the prevalence of domestic smoking, both in 2007 and 2010 (Figure 2). The lower the education of the parent, the higher was the prevalence of domestic smoking and exposure of children to SHS. In regression analyses, taking sex and age into account, we found that the lower the education of the parent the higher was the probability of smoking in the home. This was seen both in 2007 and in 2010 (Figure 3). In 2010 it was more than 11 times more likely for a child to be exposed to SHS at home if the parent had very low education than if he/she had a high education.

**Figure 4:**
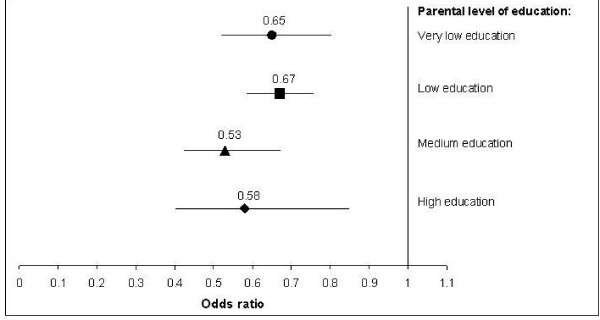
**Temporal changes from 2007 to 2010 in smoking inside homes with children by parents’ level of education.** Each level of education is compared with the same education level 3 years later. Year 2007 is reference. Analyses adjusted for age and sex.

In the sex and age adjusted regression analyses we found a significant decrease in smoking inside homes with children from the year 2007 to the year 2010, across all levels of education (Figure 4). When including smoking status in the regression analyses a slightly lower odds ratios were found, which would be expected if smoking status is an intermediate variable, but the overall results were unchanged (data not shown).

The odds of a temporal decrease in domestic smoking were a little higher in parents with medium and high education than in those with very low or low education, but the differences were not significant as there was no interaction between time and education (p=0.66). Neither was there significant interaction between time and sex (p=0.86), time and smoking status (p=0.56) or time and age (p=0.68).

## Discussion

In a repeated large population-based survey from the Capital Region of Denmark we found marked and persistent social inequalities in children’s exposure to SHS; the lower the education of the parent/adult the higher the probability of smoking in the home. There was significant temporal decrease in domestic smoking from 2007 to 2010, independent of education level. Thus, social disparities in the level of children’s exposure to SHS at home have persisted over time, but not increased significantly.

It has been documented that children’s exposure to SHS has declined over recent decades [17-19] and it is also known that there are large social disparities in children’s exposure to SHS at home. Parents with a low SES and single parents have a higher prevalence of smoking and are less likely to have implemented smoking bans at home [22,23]. However, to our knowledge, only four studies have investigated the temporal changes in social disparities in children’s exposure to SHS [18,25,26,28]. A recent Australian study found that the proportion of children who lived with a smoker had declined in all social groups except the most disadvantaged households [26]. A study from Wales found that reductions in SHS exposure were limited to children from higher SES households [25]. A Scottish study found that the introduction of smoke-free legislation had reduced exposure to SHS among all children [28]. Results also indicated that inequalities in cotinine concentration in children increased after legislation. According to an English study, however, the decline in cotinine over time tended to be greatest in children who were most exposed, indicating that absolute inequalities in exposure to SHS have fallen from 1996 to 2006 [18]. In our study, no significant increase or decrease in social inequalities over time was found. The different findings may be explained by national or temporal differences or differences in the measurement of exposure to SHS or measurement of socioeconomic status.

On August 15, 2007, a few weeks before the first health survey of the Capital Region of Denmark, a national smoking ban was implemented. Our data represents the time immediately after implementation of the legislation and three years later. The temporal reductions in smoking at home might therefore reflect an effect of the legislation, but might also reflect a secular trend towards smoke-free homes-a longer observational period before legislation would be needed to answer that. Reduction in children’s exposure to SHS, and an increase in the proportion of children reporting a ban on smoking in their household have been reported after implementation of the smoke-free legislation in several western countries [21,28-32]. However, in Hong Kong it seems that a comprehensive smoke-free legislation might have displaced smoking into the homes of children [33], which underlines the importance of strong simultaneous support for smoking cessation and comprehensive information about children’s health hazards when exposed to SHS.

Smoking outdoors with the door closed does not constitute total protection, but is the most effective way to protect children from environmental tobacco-smoke exposure. Other modes of action has been shown to have a minor effect [34]. The low levels of knowledge of the adverse health effects of SHS, especially among smokers, and the known relationship between knowledge and SHS-protective behaviours, suggest that greater efforts to educate smokers about the risks associated with SHS are worthwhile [35,36]. An important factor in reducing inequalities in SHS exposure among children lies in educating people, especially parents with less education/income, about the health benefits of keeping their homes smoke-free. It is an important task for health professionals, teachers, nursery teachers and so on, but it is difficult, expensive and time consuming. A recent Australian study reported that child health services in almost eight out of ten cases did not assess the SHS exposure of any child [37]. Many counselling interventions for parents have tried to reduce children’s exposure to SHS at home. Controlled trials of clinicians’ one time counselling services have shown null results [38]. A review by Cochrane found that in only 11 of 36 studies was there a statistically significant intervention effect, and that there was limited support for more intensive counselling interventions for parents [39]. Another review concluded that studies with more rigorous study designs, interventions of greater intensity and duration, and those based on sound behaviour change theory have yielded the most promising results [40]. Another approach, interventions to achieve cessation among parents, for the sake of the children, can help protect vulnerable children from harm due to tobacco smoke exposure. However, most parents do not quit, and additional strategies to protect children are needed [41].

Home smoking bans are surrounded by social, legal, and political challenges and so far no state or country in the world has dared to implement legislation banning smoking in homes with children. In Australia, however, in 2009, smoking was banned in cars if children are present [42]. In many countries around the world corporal punishment in the home is outlawed. Even though there is no evidence that outlawing smoking in the home will reduce SHS in the home and that this will be equal across SES it seems reasonable to suggest legislative protection of children from tobacco smoke inside their home. In general, legislation has shown to be a very strong instrument in tobacco control. Home smoking bans may also contribute towards a reduced risk of children becoming smokers, particularly when their parents smoke [43,44], thereby further reducing social disparities in smoking-related morbidity and mortality in the longer term.

Strengths of this study include the large study size, and the random sample of a general population. It is also a strength that the statistical analyses were weighted for the size of municipalities and for non-responses, which increases the generalizability of the study. Information about those co-habiting with a child/children, and about education levels, is from the National Central Registers, which have a high validity.

A limitation of the study is that information on smoking in the home is self-reported. It would have strengthened our results if we could have provided objective data on children’s exposure to SHS, e.g. via salivary cotinine measurements, but this was not possible in the large scale survey. More detailed information on where and how many persons smoked in the home would have been useful. It would have strengthened our study if we had used other measures of socioeconomic status, such as income or the employment status of the parent/adult. Measuring SES is very complex and each measurement has different strengths and weaknesses. There is no single best indicator of SES [45,46]. Level of education can be defined and applied regardless of working circumstances, it is a strong determinant of employment and income and it generally reflects knowledge [45,46]. The responding adult has, for the sake of convenience been called the parent, but in some cases it will be a step-parent, guardian, older sibling or another adult person living in a household with a child. The definition of a smoke-free home is not really a smoke-free home, as we have included ‘almost never’ and ‘less than weekly’ in our definition. Finally, other predictors for exposure, such as age of youngest child, could have been included. It has been reported that persons with infants in the home are more likely to have a smoke-free home than those with older children [35]. However, we believe this is consistent over time.

## Conclusions

In two large population-based studies from the Capital Region of Denmark we found marked social inequalities in children’s exposure to SHS. There was a significant temporal decrease in domestic smoking from 2007 to 2010, independent of parents’/adults’ education level. Huge social disparities have persisted over time, but not increased. Both clinical and population policy interventions will be needed to control children’s SHS exposure. Strong support for smoking cessation and comprehensive information on health hazards for children when exposed to SHS is needed but due to the high costs, high time consumption, and previously disappointing results from individual counselling it also seems reasonable to suggest legislative protection of children from tobacco smoke inside their home.

## Abbreviations

SHS: Second hand smoke; SES: Socio economic status.

## Competing interests

All authors state that they have nothing to declare.

## Authors’ contribution

CG, LHH and TJ contributed to conception and design of the surveys. LHH and CG gathered the data. AHA performed the statistical analyses. CP interpreted the data, drafted the article and designed the figures. All authors participated in the interpretation of results, revised the manuscript critically and approved it.

## Author details

^1^Research Centre for Prevention and Health, The Capital Region of Denmark, Glostrup, Denmark. ^2^Faculty of Health Science, University of Copenhagen, Copenhagen, Denmark. ^3^Faculty of Medicine, University of Aalborg, Aalborg, Denmark.
